# Establishing a Reference Procedure Length for Anterior Cervical Fusions: The Role for Standards in Surgical Process Improvement

**DOI:** 10.7759/cureus.22615

**Published:** 2022-02-25

**Authors:** Michael Bohl, Udaya K Kakarla, Steve W Chang, Rajiv Sethi, Farrokh Farrokhi, Jean-Christophe Leveque

**Affiliations:** 1 Neurosurgery, Carolina Neurosurgery & Spine Associates, Charlotte, USA; 2 Department of Neurosurgery, Barrow Neurological Institute, Phoenix, USA; 3 Department of Neurosurgery, Virginia Mason Medical Center, Seattle, USA

**Keywords:** anterior cervical discectomy and fusion, surgical process improvement, standard work, reference outcome measures, procedure length, clinical outcomes

## Abstract

Surgical process improvement strategies are increasingly being applied to specific procedures to improve value. A critical step in any process improvement strategy is the identification of performance benchmarks. Procedure length is a performance benchmark for anterior cervical discectomy and fusion (ACDF) procedures; therefore, we sought to establish reference procedure lengths for 1-level, 2-level, and 3-level ACDFs at both teaching and non-teaching institutions and to describe methods for using this information to advance surgical process improvement initiatives. We performed a retrospective analysis of consecutive ACDFs performed at a resident teaching institution (RT) and a non-teaching institution (NT) for all 1-level, 2-level, and 3-level ACDFs. Mean case lengths and patient outcomes were calculated for individual surgeons and institutions. After limiting cases to 1-level, 2-level, and 3-level ACDFs and applying all exclusion criteria, 991 cases at the RT institution and 131 cases at the NT institution (a total of 1122 cases) were available for analysis. The mean (SD) procedure length for 1-level, 2-level, and 3-level ACDFs at the RT versus NT institutions were 121.9 min (36.3 min) and 73.6 min (29.7 min) (p<0.001), 172.7 min (44.8 min) and 112.0 min (43.0 min) (p<0.001), and 218.3 min (54.9 min) and 167.6 min (54.2 min) (p<0.001), respectively. Thirty-day outcomes were the same between institutions, except that the RT institution had a shorter mean hospital length of stay for 2-level ACDFs (1.6 days versus 2.9 days, p=0.001). This study is the first to attempt to establish a standard reference procedure length for 1-level, 2-level, and 3-level ACDFs. These data can guide efforts in surgical process improvement.

## Introduction

As healthcare expenditures in the United States continue to rise at an unsustainable rate, the value of certain surgical procedures is being increasingly scrutinized [1]. Because of the high global prevalence of spine disease and the increasing rate of surgical treatment of the spine, many researchers have begun focusing on evaluating and improving the safety and value of spinal surgery [2-4]. Cervical spine surgeries are among the most commonly performed procedures in the United States, with an increase in the diagnosis and surgical treatment of degenerative cervical disease over the previous decade [5-7]. Continued efforts are therefore warranted to improve outcomes and reduce costs for these procedures.

Surgical case length is one of the strongest predictors of adverse outcomes and increased costs in anterior cervical discectomy and fusion (ACDF) procedures [8,9]. It is difficult to establish operative time as a truly independent predictor of outcomes because of its close relationship to the severity of pathology being addressed. However, a recent study evaluating more than 15,000 single-level ACDFs found that additional operative times of as little as 15 minutes were predictive of worse outcomes [8]. The authors of this study concluded that surgeons should maximize operative efficiency as much as safely possible, as even a 15-minute improvement in case length could lead to better outcomes. Another study has shown that case length is one of the biggest drivers of 90-day costs for elective ACDFs [9]. Efforts to reduce ACDF procedure length might therefore improve value both by reducing costs and improving outcomes.

Many studies evaluating patient outcomes after ACDF procedures report a mean procedure length for their study population, but none report these variables with enough granularity to establish a standard reference procedure length for ACDF [8-16]. For example, many studies combine 1-level, 2-level, and 3-level ACDFs (or two of the three procedure types) in their reported mean or median operative length calculation [9,11-13]. Other studies combine ACDFs and cervical disc arthroplasties in procedure length calculations [10]. Others report on 1-level ACDFs only but do not differentiate between procedures done at teaching institutions with residents and those done at non-teaching institutions [8]. The presence of residents in various neurosurgical procedures has been demonstrated to increase procedure length, though none of these studies have shown worse outcomes as a result [17-22]. In an increasingly value-driven healthcare market, it is important to establish standards to serve as a reference for future improvement efforts. The father of lean manufacturing, Taichi Ohno, perhaps put it best when he stated, “Without standards, there can be no improvement” [23]. The purpose of this study, therefore, was to establish reference procedure lengths for 1-level, 2-level, and 3-level ACDFs at both teaching and non-teaching institutions and to describe methods for using these data to advance surgical process improvement initiatives.

## Materials and methods

The Institutional Review Boards of Virginia Mason Medical Center, Seattle, Washington, and St. Joseph’s Hospital and Medical Center, Phoenix, Arizona, approved this study. We retrospectively analyzed consecutive ACDF procedures from a period spanning 35 months at two different spine centers (July 2013 to May 2016), one being a resident teaching institution (RT) and the other a non-teaching institution (NT). Surgeons at the NT institution regularly worked with a physician’s assistant whose role in an ACDF was limited to retraction and surgical site closure. Cases for inclusion were identified based on the surgeon-provided case description in the electronic medical record. Procedures that involved one or more corpectomies, hardware revisions, washouts for infection or hematoma, a second stage posterior procedure immediately preceding or following the ACDF, or disc arthroplasty were excluded from the analysis. Adjacent segment ACDFs that did not include hardware revision were included. Collected case variables included the operating surgeon, procedure length from skin incision to skin closure, patient age, the number of levels treated, whether or not the patient was admitted through the emergency department, the hospital length of stay (LOS) from skin incision to discharge, returns to the emergency department within 30 days (RED30), hospital readmissions within 30 days (RAD30), returns to the operating room within 30 days (ROR30), mortality within 30 days (MOR30), and whether hospital discharge was to home, acute rehabilitation facility, or skilled nursing facility. The decision was made not to collect more detailed patient demographics or to perform univariate or multivariate analyses based on demographics as we felt these analyses might distract from the purpose of the study, which was not to determine causes or predictors of extended ACDF procedure length, but rather set initial benchmarks for ACDF procedure length, then to describe methods for using these benchmarks to advance surgical process improvement initiatives.

Cases were divided into groups based on RT or NT institution and the number of levels treated. Mean case lengths were then calculated for each surgeon. Surgeon mean case lengths were compared graphically, and overall mean case lengths were calculated for 1-level, 2-level, and 3-level ACDFs at RT and NT institutions.

Statistical comparisons were then performed between RT and NT institutions. Age and number of cases admitted through the emergency department were compared using Student t-tests and chi-square analysis with Fisher exact probability tests. To determine whether a difference existed between institutions for short-term adverse events, hospital LOSs were compared using student t-tests, and RED30, ROR30, RAD30, MOR30 (30-day adverse events) and disposition at discharge were compared using chi-square analyses and Fisher exact probability tests. The significance level was set at p<0.05.

To determine whether longer cases had worse outcomes, all cases were divided into two groups: those that were less than 1 SD above the mean case duration for each physician (regular case length) and those that were greater than 1 SD above each surgeon’s mean case duration (extended case length). Assuming a normal distribution, this would result in a comparison of the longest 16% of cases for each surgeon against all shorter cases for that surgeon. Hospital LOS was then compared between regular case length cases and extended case length cases using Student t-tests. All categorical adverse events were then grouped to perform a chi-square comparison between regular case length and extended case length groups for 1-level, 2-level, and 3-level ACDFs. Finally, the surgeons in the study with the highest case volumes were analyzed using the same statistical comparisons between regular case length and extended case length groups. We defined the cut-off for high volume caseload at 100 total cases or more included in the analysis. Lower volume surgeons were excluded from this analysis because they lacked sufficient case numbers to provide a meaningful statistical comparison between groups.

## Results

A total of 1309 cases were identified at the RT institution and 164 cases at the NT institution during the 35-month study period, for a total of 1473 cases. After limiting the cases to 1-level, 2-level, and 3-level ACDFs and applying all exclusion criteria, 991 cases at the RT institution and 131 cases at the NT institution (a total of 1122 cases) remained for analysis. The RT institution included 13 different attending neurosurgeons, all operating with neurosurgical residents for each case. The mean post-graduate year for all cases combined was 3.7 (range, 1-8). The NT institution included seven different attending surgeons comprising six neurosurgeons and one orthopedic surgeon. Table 1 shows analyzed cases by institution type and the number of ACDF levels.

**Table 1:** Cases analyzed by institution and the number of cervical levels ACDF: anterior cervical discectomy and fusion. NA: not applicable.

Group	No. Cases	No. Surgeons	Resident Post-Graduate Year, Mean (Range)
RT Institution			
1-level ACDFs	403	13	3.9 (1-8)
2-level ACDFs	355	13	3.7 (1-8)
3-level ACDFs	233	13	3.6 (1-8)
NT Institution			
1-level ACDFs	66	6	NA
2-level ACDFs	47	6	NA
3-level ACDFs	18	3	NA
Total	1122	20	3.7 (1-8)

**Figure 1:**
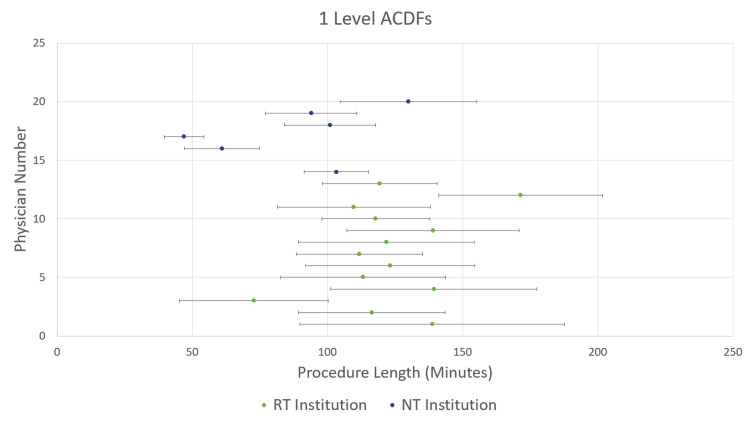
Mean procedure lengths for 1-level anterior cervical discectomy and fusion (ACDF) procedures by individual physician. Data are for individual physicians numbered from 1 through 20 on the y-axis; the RT group is numbered 1 through 13, and the NT group is numbered 14 through 20. Bars show standard deviations.

**Figure 2:**
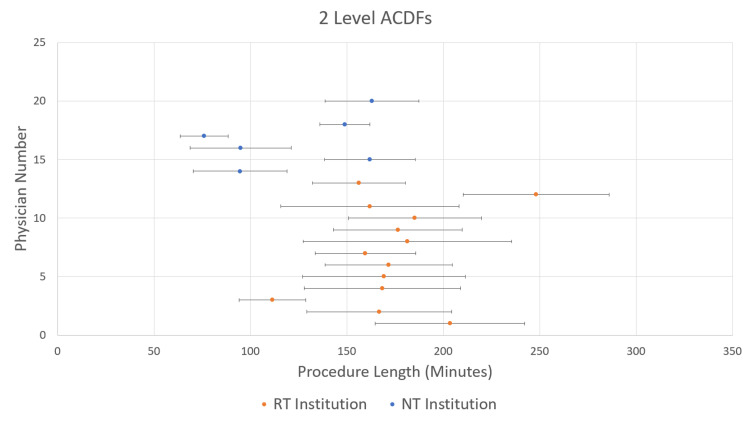
Mean procedure lengths for 2-level anterior cervical discectomy and fusion (ACDF) procedures by the individual physician Data are for individual physicians numbered from 1 through 20 on the y-axis; the RT group is numbered 1 through 13, and the NT group is numbered 14 through 20. Bars show standard deviations.

**Figure 3:**
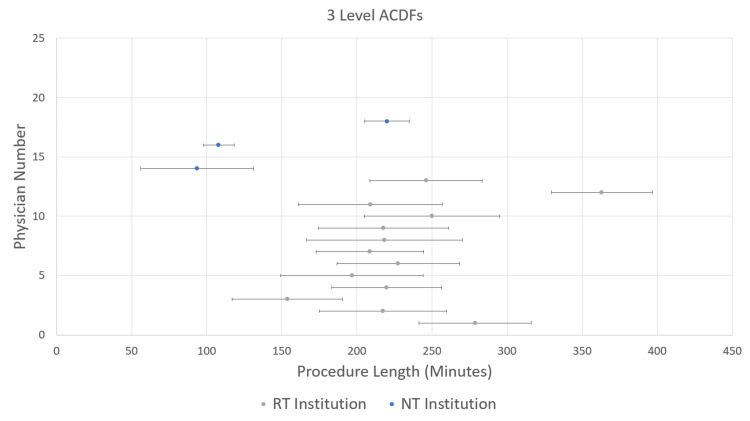
Mean procedure lengths for 3-level anterior cervical discectomy and fusion (ACDF) procedures by the individual physician Data are for individual physicians numbered from 1 through 20 on the y-axis; the RT group is numbered 1 through 13, and the NT group is numbered 14 through 20. Bars show standard deviations.

The mean (SD) case lengths for 1-level ACDFs were 121.9 min (36.3 min) at the RT institution and 73.6 min (29.7 min) at the NT institution (p<0.001) (Figure 1). For 2-level ACDFs, the mean (SD) case lengths were 172.7 min (44.8 min) at the RT institution and 112.0 min (43.0 min) at the NT institution (p<0.001) (Figure 2). Finally, for 3-level ACDFs, the mean (SD) case lengths were 218.3 min (54.9 min) at the RT institution and 167.6 min (54.2 min) at the NT institutions (p<0.001) (Figure 3). Mean and single SD ranges were then calculated for each surgeon and plotted on the same graphs (Figures 1-3). As can be seen in Figures 1-3, the majority of surgeons in the RT institution fall within the same range of procedure length. The surgeons in the NT institution appear to be evenly divided between two groups.

Case variables and 30-day outcomes were then evaluated and compared between institutions. There were no significant differences between RT and NT institutions for the number of cases admitted through the emergency department, RED30, ROR30, RAD30, MOR30, or discharge to home, acute rehabilitation center, or skilled nursing facility for all 1-level, 2-level, and 3-level ACDFs. For 1-level and 2-level ACDFs, there were significant differences between RT and NT institutions in the mean patient age, but the size of difference does not appear to be clinically significant (54.4 years versus 60.8 years, p<0.001; 56.3 years versus 62.1 years, p=0.001). For 2-level ACDFs, patients treated at the RT institution had significantly shorter hospital LOSs (1.6 days versus 2.9 days, p=0.001). Table 2 shows comparisons of outcomes between the RT and NT institutions (Table 2). MOR30 data comparisons are excluded as there were no cases of 30-day mortality in the entire population of analyzed patients.

**Table 2:** Statistical comparison of case variables and outcomes between institutions Admit: admittance; ED: emergency department; LOS: hospital length of stay; NT: non-teaching institution; RAD30: hospital readmission in 30 days; RED30: returns to the emergency department in 30 days; Rehab: rehabilitation; ROR30: return to the operating room in 30 days; RT: resident teaching institution; SD: standard deviation; SNF: skilled nursing facility. Boldface type indicates significant values (p<0.05).

Groups	No. Cases	Patient Age, Mean (years)	Procedure Length, Mean (SD), min	ED Admit, %	LOS, days	RED30, %	ROR30, %	RAD30, %	Type of Facility Discharged to		
									Home, %	Acute Rehab, %	SNF, %
1-Level											
RT	403	54.4	121.9 (36.3)	15.6	1.7	7.4	1.5	3.2	89.6	4.5	3.5
NT	66	60.8	73.6 (29.7)	13.6	1.6	3.0	1.5	1.5	95.5	0	4.5
p-value	-	<0.001	<0.001	0.719	0.865	0.207	1.000	0.704	0.176	0.091	0.719
2-Level											
RT	355	56.3	172.7 (44.8)	9.3	1.6	8.7	1.7	3.9	90.7	3.9	2.8
NT	47	62.1	112.0 (43.0)	14.9	2.9	4.3	4.3	4.3	89.4	6.4	4.3
p-value	-	0.001	<0.001	0.295	0.001	0.403	0.238	1.00	0.790	0.434	0.639
3-Level											
RT	233	58.3	218.3 (54.9)	9.4	2.5	8.2	1.7	5.2	91.0	4.7	2.6
NT	18	57.2	167.6 (54.2)	5.6	3.1	11.1	0	0	94.4	0	5.6
p-value	-	0.653	<0.001	0.712	0.542	0.457	1.000	0.610	1.000	0.615	0.410

Comparison of outcomes between each surgeon’s normal length and extended length procedures demonstrated significantly longer hospital LOSs for extended length 1-level ACDFs (1.5 days versus 2.5 days, p=0.004) and 2-level ACDFs (1.5 days versus 2.2 days, p=0.013) at the RT institution, and significantly longer hospital LOSs following extended length 1-level ACDFs at the NT institution (1.23 days versus 3.3 days, p=0.006). All other comparisons of outcomes were not significant, though nearly all trended toward worse outcomes for extended-length procedures. (Table 3).

**Table 3:** Comparison of outcomes between regular length and extended length cases LOS: hospital length of stay; NT: non-teaching institution; RT: resident teaching institution. Boldface type indicates significant values (p<0.05).

Outcomes	RT Institution			NT Institution		
	Regular Length	Extended Length	p-value	Regular Length	Extended Length	p-value
LOS, mean (days)						
1-Level	1.47	2.50	0.004	1.27	3.33	0.006
2-Level	1.53	2.18	0.013	2.04	2.4	0.774
3-Level	2.39	3.19	0.230	2.54	1.37	0.521
30-Day adverse events, (% incidence)						
1-Level	9.3%	7.1%	0.636	3.4%	0%	0.999
2-Level	7.6%	9.7%	0.605	5.1%	12.5%	0.999
3-Level	7.6%	16.7%	0.108	13.3%	0%	0.999

Finally, four surgeons met the criteria for high case volume (100 or more qualifying cases during the study period). All four surgeons were from the RT institution, and together they accounted for 53.7% of the total analyzed case volume. Comparison of regular length and extended length cases for each of these surgeons individually did not find significant differences in any of the measured outcomes (Table 4).

**Table 4:** Comparison of outcomes between regular length and extended length cases by individual surgeon LOS: hospital length of stay.

		LOS, days			30-Day Adverse Events, %		
Surgeon	No. Cases	Regular Length	Extended Length	p value	Regular Length	Extended Length	p-value
1	183	1.5	2.5	0.154	7.6%	15.4%	0.251
2	167	2.0	2.8	0.123	8.5%	14.8%	0.471
3	134	1.9	2.7	0.161	10.6%	4.3%	0.469
4	119	1.5	1.8	0.719	4.9%	11.8%	0.258

## Discussion

Data interpretation

**Figure 4:**
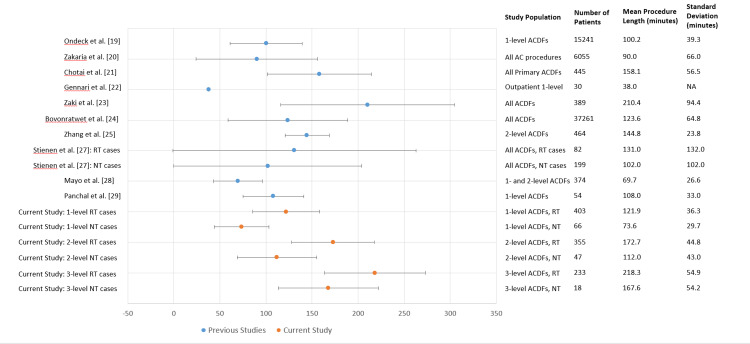
Previously published mean ACDF procedure lengths Mean anterior cervical discectomy and fusion (ACDF) procedure lengths and case characteristics in previously reported studies compared to the mean procedure lengths and case characteristics reported in this study. Bars show standard deviations.

This is the first study to establish a reference procedure length for 1-level, 2-level, and 3-level ACDFs at both RT and NT institutions. We hope that these data will be used as an initial benchmark upon which future system process improvement studies can be based. Many studies have reported mean or median procedure lengths for heterogeneous populations of patients undergoing a variety of anterior cervical procedures [8-13,15,16]. Figure 4 provides a summary of these studies with population characteristics and reported procedure lengths compared to our results (Figure 4) [8-13,15,16]. As can be seen from these data, previously published literature on ACDF procedure lengths are difficult to generalize because they are reported either as aggregate means, which are inclusive of all numbers of levels and types of anterior cervical procedures, or they are reported as single surgeon experiences with 1-level or 2-level ACDFs. These limitations make it difficult to determine a useful reference procedure length for ACDFs. Figure 4 also demonstrates that the values obtained in this study are within the same range as other studies have reported, suggesting this study’s procedure lengths are reliable as references (Figure 4). The homogeneity of the study populations presented here, together with the number of physicians evaluated, suggests these data are reliable as an initial reference for 1-level, 2-level, and 3-level ACDFs at RT and NT institutions. Future studies on systems process improvement methods for ACDF procedures will hopefully use these data and provide newer, updated reference procedure lengths as iterative process improvements are made.

The statistical comparisons of procedure length and patient outcomes between RT and NT institutions demonstrate that procedures at the RT institute took significantly longer than procedures at the NT institute. However, the data showed either no difference in outcomes or better outcomes for the RT patients (LOS for 2-level ACDFs) than the NT patients. These results contradict the conclusions of some studies that demonstrate worse outcomes for cases that take longer but are consistent with several studies that evaluated the effect of increased resident involvement on short-term outcomes [8,17-22]. For example, Ondeck et al. (2018) presented a database review of 15,241 patients and found that the length of the ACDF procedure is associated with an increased rate of adverse events. Our data do not show a correlation between procedure length and short-term outcomes. There are several potential reasons for this discrepancy. First, the objective of our study was vastly different from that of Ondeck et al. (2018). Our study attempts to establish reference procedure lengths for 1-level, 2-level, and 3-level ACDFs and to define methods for using these data for individual and institutional surgical process improvement initiatives. As such, our sample sizes, variables collected, study methods, and data analyses are completely different. With regards to the impact of resident involvement, several previous studies that have evaluated the impact of increased resident involvement in neurosurgical procedures, including spine procedures, have demonstrated that cases involving residents take significantly longer, but short-term outcomes are the same or improved compared to cases with less resident involvement [17-22]. The current study contributes to this growing body of evidence on the net neutral, or even slightly positive, the impact of resident surgical education on surgical outcomes by demonstrating that this trend holds true in inter-institutional comparisons between RT and NT programs.

Using a reference procedure length in surgical process improvement

In an increasingly value-driven healthcare market, it is important to establish meaningful metrics of work process efficiency because these metrics will serve as references for future improvement efforts. Many institutions have adopted lean manufacturing principles to guide their efforts toward establishing standard work processes aimed at improving the value of surgical spine care [23-28]. One of the central tenets of using lean methodologies to create value in complex systems (like those involved in the delivery of surgical spine care) is to establish a standard work process. By establishing a standard work process, one is better able to determine which steps of that process are contributing value to the overall work and which are producing waste. By establishing a reference procedure length for ACDFs, individual physicians and institutions will have a starting benchmark for comparison of their future work on process improvement. This is not to say that other, potentially better measures of surgical process efficiency besides procedure length should not be measured. On the contrary, a truly robust standard work process is reliant on identifying all meaningful measures of work process value and continuously monitoring those outcomes. Procedure length is simply among the easiest to measure and perhaps most indicative of overall process efficiency. Procedure length, therefore, seems as good a starting point as any for establishing a baseline standard reference for continuous work process improvement efforts.

The purpose of this study is not to claim that the institutions and surgeons involved should be considered the standard-bearers for ACDF outcomes or procedure lengths. Rather, by performing this analysis, we now have a methodology and a reference measure of surgical process efficiency (procedure length) that can be monitored when modifying our practices in hopes of achieving greater operational efficiency and value in the future. The benchmark procedure lengths reported in this study are not intended to be considered the standard to which others should strive. On the contrary, they are simply meant to provide a starting point upon which any future studies on ACDF process improvement can be based. It is our hope and intention that the procedure length benchmarks in this study are challenged and revised, as this would indicate the presence of active and robust system process improvement efforts in our field.

Of course, faster surgeries are not by any means better surgeries, and slow surgeries are not necessarily associated with worse outcomes. The purpose of this study is also not to punitively identify physicians operating at either end of the procedure length bell curve but rather to give all physicians information that they can use to guide their continued development and improvement in surgical technique and efficiency. It is difficult for a surgeon to know how to improve without first knowing where he or she falls on the bell curve, and it is impossible without first knowing where one’s performance lies. For example, a surgeon at an RT institution who is operating twice as fast as the next fastest surgeon might consider spending more time teaching. Similarly, a surgeon operating more slowly than his/her colleagues may consider identifying a fellow surgeon who is close to them on the bell curve to discuss differences in their surgical approaches. These benchmarks provide physicians a means to define their relative performance and to identify colleagues who might be useful to engage in mutual coaching efforts.

Establishing a benchmark can also enable us to work towards defining acceptable procedure lengths. For example, it would likely be worthwhile to discuss with any surgeon who regularly falls greater than two standard deviations outside the mean procedure length what they are or are not doing that results in exceptionally long or short cases. In addition, institutions might find this information useful in the development of early warning systems. Longer procedure times appear to be associated with longer postoperative hospital stays and potentially more short-term adverse events. If an institution knows an individual surgeon’s standard case length for any individual procedure, then during procedures that appear to be taking longer than a standard deviation from the mean, the hospital could intervene early to offer more resources to the attending surgeon (e.g., by providing a surgical colleague to ask if any help is needed, additional hospital staff to help retrieve needed instruments or technical personnel to determine if equipment failure is contributing to the longer than expected procedure length). This type of early warning system would be of value to institutions attempting to standardize operative workflow and efficiency and is consistent with a lean approach to standardizing perioperative patient care.

## Conclusions

The father of standard work, Taichi Ohno, famously said, “Without standards, there can be no improvement." This is the first study to attempt to establish a standard reference procedure length for 1-level, 2-level, and 3-level ACDFs at RT and NT institutions. One of the most critical steps in establishing standard work processes is to identify standard outcome measures of performance against which all future efforts toward improvement can be compared. In the spirit of continuous self-improvement, we intend to use these data to guide our individual and institutional efforts toward better operative quality and efficiency. We hope that other physicians and institutions will use these references and this lean methodology to further improve their work processes for ACDF procedures and that any institution that achieves superior performance will similarly share its data and quality improvement methodology so that we and others may learn and benefit from it.
